# Efficacy, safety and tolerability of escitalopram in doses up to 50 mg in Major Depressive Disorder (MDD): an open-label, pilot study

**DOI:** 10.1186/1471-244X-11-42

**Published:** 2011-03-16

**Authors:** Alan G Wade, Gordon M Crawford, Ann Yellowlees

**Affiliations:** 1 CPS Research, Glasgow, G20 0XA, UK; 2 Quantics Consulting Limited, Tweed Horizons, Newtown St Boswells, Scottish Borders TD6 0SG, UK

## Abstract

**Background:**

Escitalopram is licensed for use at doses up to 20 mg but is used clinically at higher doses. There is limited published data at higher doses and none in the treatment of Major Depressive Disorder (MDD).

**Methods:**

This open-label, pilot study was designed to investigate the efficacy, safety and tolerability of escitalopram in doses up to 50 mg in MDD. It was conducted in 60 primary care patients with MDD who had not responded to adequate treatment with citalopram. Patients were treated with escalating doses of escitalopram up to 50 mg for up to 32 weeks until they achieved remission (Montgomery-Asberg Depression Rating Scale [MADRS] ≤8) or failed to tolerate the dose.

**Results:**

Forty-two patients (70%) completed the study. Twenty-one patients (35%) achieved remission with 8 of the 21 patients (38%) needing the 50 mg dose to achieve remission. Median time to remission was 24 weeks and median dose in remission was 30 mg. No significant safety issues were identified although tolerability appeared to decline above a dose of 40 mg with 26% of patients unable to tolerate 50 mg. Twelve (20%) patients had adverse events leading to discontinuation. The most common adverse events were headache (35%), nausea, diarrhoea and nasopharyngitis (all 25%). Minor mean weight gain was found during the study, which did not appear to be dose-related. Half of the patients who completed the study chose to continue treatment with escitalopram rather than taper down the dose at 32 weeks.

**Conclusions:**

Dose escalation with escitalopram above 20 mg may have a useful role in the management of patients with MDD, although further studies are needed to confirm this finding.

**Trial Registration:**

ClinicalTrials.gov: NCT00785434

## Background

Selective serotonin reuptake inhibitors (SSRIs) are considered a first-line pharmacological treatment for major depressive disorder (MDD). However, up to 50% of patients may fail to respond to the initial treatment and as few as 30% achieve the treatment goal of full remission [1]. Remission has been defined at different levels of the MADRS scale, but for the purposes of this study we used the value of <9 as representing relative "wellness" [2]. Three major strategies are recommended by the guidelines to manage these patients: dose escalation, augmentation and switching to another antidepressant of the same or a different class. Dose escalation is often the first choice of clinicians, although there is limited evidence to support this strategy.

Citalopram is one of the most commonly used antidepressants in the United Kingdom and was selected for study to standardise and simplify recruitment to the study. However, citalopram is a racemic mixture of the R- and S-enantiomers in a 1:1 ratio, with only the S-enantiomer (escitalopram) associated with antidepressant activity. It is thought that the R-enantiomer competes with the S-enantiomer at a low-affinity site on the serotonin reuptake transporters (SERTs), leading to decreased binding of the S-enantiomer at the high-affinity site [3]. Therefore, increasing the dose of citalopram may not lead to better efficacy of the S-enantiomer due to increasing interference from the R-enantiomer [4].

Escitalopram, uniquely among the SSRIs, potentiates its own binding, raising the possibility of increasing effect with increasing doses [5]. In the United Kingdom, it is currently licensed for the treatment of major depressive episodes at doses of 5 mg, 10 mg and 20 mg [6]. There is evidence from marketing data and anecdotal reports that clinicians are using escitalopram at doses considerably higher than the recommended maximum of 20 mg. However, there is little published literature available to support the use of escitalopram at these higher doses and none in the treatment of MDD [7].

The relationship between SSRI starting dose and treatment outcome in MDD has been examined recently in a meta-analysis [8]. This indicated that patients receiving the usual starting dose of SSRIs (such as 10 mg escitalopram) were less likely to respond than patients who received higher starting doses. However, starting treatment with higher doses of SSRIs was associated higher rates of discontinuation due to intolerance.

The objectives of this open-label, pilot study were to investigate the efficacy, safety and tolerability of escitalopram in doses up to 50 mg in the treatment of MDD.

## Methods

### Study design

This was an open, pilot study of escitalopram in patients with MDD who had not responded to treatment with citalopram (clinicaltrials.gov identifier: NCT00785434). Patients meeting the entry criteria for the study were recruited by the General Practitioner (GP) and thereafter managed in conjunction with a CPS research assistant. Regardless of citalopram dose, patients were switched abruptly to escitalopram 10 mg and treated with 2-weekly escalating doses of escitalopram up to a maximum of 50 mg for up to 32 weeks (Table 1) until they either achieved remission according to the Montgomery-Asberg Depression Rating Scale [9] (MADRS ≤8) or failed to tolerate the dose. Thereafter, patients who achieved remission were maintained on the remission dosage and reviewed at 4 weekly intervals. At any subsequent visit where the MADRS score was >8, the dosage was increased. Patients unable to tolerate a dose had their dosage reduced to the previous tolerable dose.

**Table 1:** Dose schedule

Visit weeks	MADRS score	**Dose of escitalopram**^**a**^
Week 1	Any	10 mg
Week 2	Any	20 mg
Week 4	Any	20 mg (review visit)
Week 6	<12	Maintain dose at 20 mg
	≥12	Increase dose to 30 mg
Week 8	≤8	Maintain current dose (20 or 30 mg) for 4 weeks
	>8	Increase dose (20 mg to 30 mg or 30 mg to 35 mg) for 2 weeks^b^
4-weekly intervals until Week 32	≤8	Maintain current dose for 4 weeks
	>8	Increase dose (20 mg to 30 mg or 5 mg increment at 2-weekly intervals) up to 50 mg^b^

^a ^Irrespective of their MADRS score, patients unable to tolerate a higher dose had their dose decreased (30 to 20 mg or 5 mg decrease). Patients with a MADRS score >8 who were intolerant of a higher dose were maintained on their current dose for 2 or 4 weeks, depending on their visit schedule.

^b^Patients with a dose increase were assessed at an additional visit 2 weeks later. At this visit, patients with a MADRS ≤8 had their dose maintained for a further 2 weeks, whereas patients with a MADRS >8 had their dose increased for 2 weeks.

Efficacy, safety and tolerability were assessed at 12 clinic visits over the 34-week study: a baseline visit at day 1, visits at weeks 1, 2, 4, 6, 8 and every 4 weeks until 32 weeks and a follow-up visit 2 weeks after starting tapering down at 32 weeks or at discontinuation. There was also a safety follow-up 28 days after the 32-week visit, which was generally performed over the telephone.

Patients were advised to taper down the doses (50 mg to 40 mg, 40/45 mg to 30 mg, 30/35 mg to 20 mg and 20/25 mg to 10 mg) at the 32-week or discontinuation visit and then to lower the dose by 10 mg every 3 days until they stopped taking escitalopram. After this, patients were managed at the discretion of their supervising physician.

The investigation was conducted in accordance with the Declaration of Helsinki and Good Clinical Practice guidelines although a non-conformance in the medication packaging was recorded which independent review indicated had no influence on the study results.

### Patient population

The study was conducted using a network of GPs based in central and west Scotland. Patients aged 18-65 years old diagnosed by the GP with MDD as defined by DSM IV criteria [10], who had shown an inadequate response to a primary course of citalopram 20 mg or greater for a minimum of 6 weeks were eligible for this study. An inadequate response was defined as failure to achieve a MADRS score of ≤12. Exclusion criteria included significant other psychiatric disorders that would interfere with trial assessments (co-morbid generalized anxiety disorder and panic disorder were allowed if MDD was considered the primary diagnosis), history of mania or bipolar disorder, known contraindication for the use of citalopram or escitalopram, significant bleeding disorder and prominent suicidal ideation (score >4 in the MADRS item 10 (suicidal thoughts)). Patients with any alcohol or substance dependence in the past 6 months, major physical illness, significant ECG, hepatic or renal liver abnormalities, pregnant or breastfeeding women and those using inadequate contraception were also excluded.

The clinical study protocol was approved by the relevant ethics committees and written informed consent was obtained from each patient prior to enrolment into the study.

### Outcome measures: Efficacy

The primary endpoint was the number (%) of patients achieving remission, where remission was defined as a MADRS total score of ≤8. MADRS remission was chosen as the primary variable as it was generally used to assess patient outcome in clinical trials of escitalopram.

Secondary outcome measures included the number (%) of patients achieving absolute sustained remission (reaching a MADRS score of ≤8 and staying at ≤8), sustained remission (reaching a MADRS score of ≤8 and staying at ≤12) and response (achieving a 50% decrease in MADRS from baseline MADRS score), mean changes in the MADRS scores from baseline and changes in Clinical Global Impressions - Improvement of Illness (CGI-I) scale [11].

### Outcome measures: Safety and tolerability

Safety and tolerability outcomes were assessed from adverse events (AEs), vital signs, weight, physical examination and ECG findings, concomitant medication, full blood count, liver function tests and electrolytes. A Discontinuation Emergent Signs and Symptoms (DESS) scale was used at the 32 week or discontinuation visit and at a follow up visit 2 weeks later to assess withdrawal symptoms [12].

### Statistical methods

The primary endpoint for the study was the proportion of patients enrolled who achieved remission at the end of the study. In order that this proportion could be estimated to within approximately ±10% (based on an approximate two sided, 90% confidence interval), 60 patients were enrolled. The 90% confidence level was used to summarise the primary endpoint for consistency with the protocol with both sides of the interval presented for completeness. All other confidence intervals were presented as two sided, 95% intervals.

Safety and tolerability data were summarised descriptively for the safety population, which included all patients who took at least one dose of the study drug. Descriptive analyses for efficacy were performed using both observed cases (OC) and baseline observation carried forward (BOCF) approaches. Efficacy outcome variables were summarised for the 'completer' population, which consisted of all patients in the safety population who reached the final study visit at 34 weeks and for all patients completing a visit, where appropriate.

Logistic regression analyses were performed to assess the relationship between age group, gender and history of anxiety and the probability of achieving remission at the end of the study. A repeated measures analysis of covariance (ANCOVA) was carried out to assess the effects over time on MADRS score. Chi-squared tests were carried out to test for association between remission at the end of the study and achieving a 50% reduction in MADRS at 8 weeks from start of treatment.

## Results

### Patients

#### Patient disposition

Sixty patients were enrolled into the study and took study medication. Forty-two patients (70%) completed the study and the main reason for treatment discontinuation was AEs. Of the 18 patients who did not complete treatment, 6 patients discontinued due to an AE in the first 2 weeks of treatment. However, the rate of discontinuation due to an AE was low for patients continuing on medication after this period (Table 2).

**Table 2:** Timing and dose at treatment discontinuation

Dose of escitalopram	Week of last completed visit	Number of patients discontinued	
		
		**Due to an AE**^**a**^	**Not due to an AE**^**a**^
10 mg	Week 0 (baseline)	6	0
	Week 8	1	0
20 mg	Week 4	1	0
	Week 16	1	0
30 mg	Week 8	1	0
35 mg	Week 8	0	2 (1 - ineligible to continue, 1 - lack of efficacy)
40 mg	Week 24	1	0
45 mg	Week 24	1	1 (lack of efficacy)
50 mg	Week 16	1	0
	Week 20	0	1 (lost to follow up)
	Week 24	0	1 (lack of efficacy)

^a ^AE: adverse event. The reason for withdrawal was taken from the End of Study Case Report Form (CRF) page. This stated that a patient withdrew due to an AE, although this was not recorded as a withdrawal as a result of an AE on the AE CRF page

#### Baseline characteristics

Demographic and clinical characteristics are presented in Table 3. The study population had a mean age of 43.5 years, a mean BMI of 30.8 and an unusually high proportion (87%) were women. Forty-two (70%) patients had a previous psychiatric history, with 3 (5%) patients having anxiety symptoms present at the baseline visit (latter result not shown).

**Table 3:** Patient baseline demographic and clinical characteristics

Characteristic	n = 60
Female, n (%)	52 (86.7%)
White, n (%)	60 (100%)
Age, mean (SD), years	43.5 (10.7)
BMI, mean (SD), kg/m^2^	30.8 (7.9)
Duration of current MDD episode, mean (SD), months ^a^	18.9 (20.2)
Other psychiatric history, n (%)	42 (70.0%)
MADRS score, n (%)	
13 - 20	15 (25.0%)
21 - 25	14 (23.3%)
26 - 30	19 (31.7%)
≥31	12 (20.0%)
MADRS score, mean (SD)	25.7 (6.31)
CGI-S score, n (%)	
3 - Mildly ill	1 (1.7%)
4 - Moderately ill	32 (53.3%)
5 - Markedly ill	22 (36.7%)
6 - Severely ill	5 (8.3%)
7 - Among the most extremely ill patients	0 (0.0%)

^a ^n = 59

#### Protocol deviations

Three patients who discontinued treatment prematurely had minor protocol deviations (2-time between visits out of the specified range, 1-patient not prescribed study medication due to a hospital admission). In addition, 22 patients who completed the study did not taper down the dose of escitalopram after the week 32 visit, but opted to continue taking the medication.

### Efficacy

#### MADRS remission

**Figure 1:**
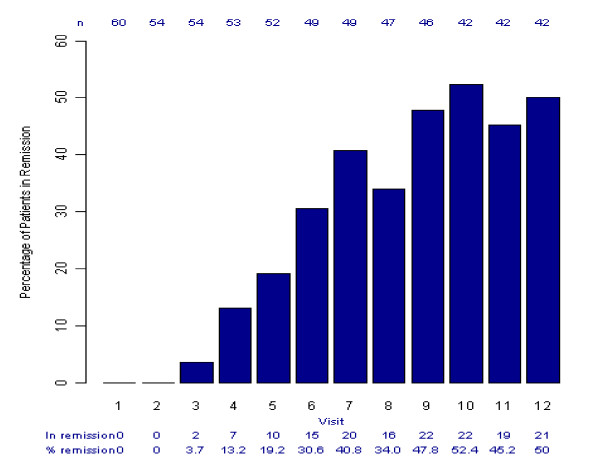
**Percentage of patients in remission per visit, all patients (observed cases per visit)**. Visits (Week) 1 (0), 2 (2), 3 (4), 4 (6), 5 (8), 6 (12), 7 (16), 8 (20), 9 (24), 10 (28), 11 (32) and 12 (34)

The number (%) of patients in remission at each study visit is shown in Figure 1 (all patients, OC). Twenty-one (35%, 90% CI 25% to 45%) of the 60 patients enrolled completed the 34-week study and achieved remission by the end of the study. This represents 50% (90% CI 37% to 63%) of the 42 patients who completed the study. One further patient achieved remission at week 6 but then discontinued due to an AE (lethargy).

Of the 21 patients who completed the study and achieved remission, 14 (67%) achieved sustained remission (achieving a score of ≤8 and staying at ≤12) and 12 (57%) achieved absolute sustained remission (achieving a score of ≤8 and staying at ≤8). The patients achieved remission over the range of 20 to 50 mg doses, with 8 (38%) of the patients requiring the 50 mg dose to reach this status (Table 4). The median time to remission was 24 weeks (range 4-34 weeks). At remission, 19 (90.5%) patients were 'very much improved' and 2 (9.5%) were 'much improved' according to the CGI-I scale.

**Table 4:** Characteristics of patients in remission (n = 21)

Characteristic	
Time to Remission (weeks)	
Mean (SD)	21.1 (12.7)
Median	24
Range	4, 34

Time in Absolute Sustained Remission (weeks)^a^	
Mean (SD)	22.5 (7.5)
Median	25
Range	6, 30

Dose at Remission^b^	
20 mg, n (%)	5 (23.8)
30 mg, n (%)	2 (9.5)
35 mg, n (%)	4 (19.0)
40 mg, n (%)	2 (9.5)
50 mg, n (%)	8 (38.1)

Dose at the 32-week visit (prior to tapering)	
20 mg, n (%)	5 (23.8)
30 mg, n (%)	3 (14.3)
35 mg, n (%)	4 (19.0)
40 mg, n (%)	1 (4.8)
50 mg, n (%)	8 (38.1)

Dose whilst in remission, mg^c^	
Mean (SD)	30.7 (11.0)
Median	30
Range	10, 50

^a ^Dose at remission is taken as the dose at first achieving absolute sustained remission for patients achieving absolute sustained remission, and the dose at the 32-week visit for those patients not achieving absolute sustained remission.

^b ^Time in absolute sustained remission is taken as the difference between the 34-week visit and the week of first achieving absolute sustained remission for patients achieving absolute sustained remission, and as 0 weeks for patients not achieving absolute sustained remission.

^c ^The dose of all episodes of remission i.e. a MADRS score of ≤8

#### Characteristics of patients by remission status

The remission status of patients completing the study is shown in Table 5 by gender, history of anxiety and age group. Logistic regression analyses indicated that women, older patients (≥45 years) and patients with no history of anxiety may be associated with a higher probability of achieving remission, but none of these associations reached statistical significance (Table 5). For example, the odds of achieving remission were approximately 4 times higher in those who had not previously experienced anxiety than those who had (odds ratio 4.10, 95% CI 0.89 to 18.89, p = 0.095).

**Table 5:** Remission Status by gender, age and history of anxiety and logistic regression analysis to determine the relationship with probability of remission, completers (n = 42)

	Total in remission (MADRS ≤8)	Total not in remission (MADRS >8)	Odds ratio (95% CI)	P-value
N (% of 42)	21 (50)	21 (50)		

Gender				
Female, n (% of n = 39)	20 (51.3)	19 (48.7)		
Male, n (% of n = 3)	1 (33.3)	2 (66.7)	3.33 (0.24-45.76)	0.350

History of anxiety				
No, n (% of n = 13)	9 (69.2)	4 (30.8)		
Yes, n (% of n = 29)	12 (41.4)	17 (58.6)	4.10 (0.89-18.89)	0.095

Age group				
Aged <45, n (% of n = 22)	9 (40.9)	13 (59.1)		
Aged ≥45, n (% of n = 20)	12 (60.0)	8 (40.0)	0.44 (0.12-1.63)	0.189

Odds ratios for female vs. male, aged <45 vs. ≥45, no history of anxiety vs. no history of anxiety from logistic regression analysis

#### MADRS Mean Score and Response

**Figure 2:**
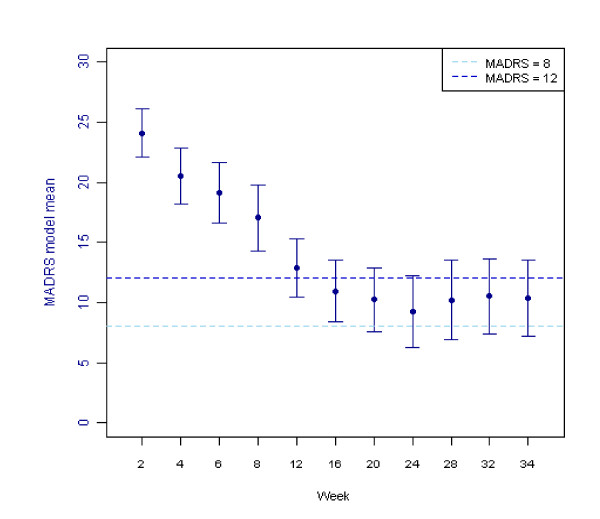
**MADRS scores over time, completers (n = 42)**. The fitted mean and 95% confidence intervals were estimated using a repeated measures analysis of covariance (ANCOVA). The fitted model included terms for time, gender age group and history of anxiety as factors, with the baseline Montgomery-Asberg Depression Rating Scale (MADRS) score and baseline body mass index (BMI) fitted as covariates.

The mean MADRS scores over time, estimated using an ANCOVA model, are shown in Figure 2 for all patients completing the study (n = 42).

**Figure 3:**
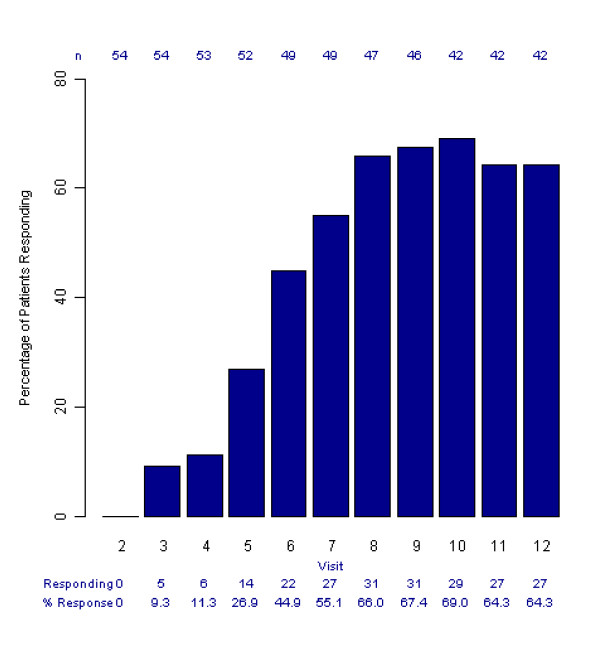
**Percentage of patients responding per visit, all patients (observed cases per visit)**. Response was defined as a 50% decrease in MADRS from baseline MADRS score. Visits (Week) 1 (0), 2 (2), 3 (4), 4 (6), 5 (8), 6 (12), 7 (16), 8 (20), 9 (24), 10 (28), 11 (32) and 12 (34)

MADRS responders (patients with at least a 50% decrease in MADRS from baseline MADRS score) at each study visit are shown in Figure 3 (all patients, OC). A chi-squared test indicated that there was a significant association between remission at the end of the study and a 50% reduction in MADRS at 8 weeks when patients were receiving either 20 or 30 mg of escitalopram (11/14, 78.6% responders in remission, odds ratio 6.60, 95% CI 1.48 to 29.36, p = 0.009).

### Safety and tolerability

Escitalopram was generally well tolerated at doses of 10 to 35 mg. Doses of 40 and 50 mg were less well tolerated with 26% of patients unable to tolerate the 50 mg dose (Table 6). However, 8 (38%) of the 21 patients who achieved remission had received the 50 mg dose.

**Table 6:** Number (%) of patients intolerant of each dose, safety population (n = 60)

Dose (mg)	**Number of patients on this dose at any time, with data available on tolerability**^**a**^	Number (% of n) of patients intolerant of this dose at any time
10	55	1 (1.8)
20	54	3 (5.6)
30	47	2 (4.3)
35	41	3 (7.3)
40	34	1 (2.9)
45	32	4 (12.5)
50	31	8 (25.8)

^a ^Reduced numbers are as a result of patient withdrawals. Additionally, some patients titrated more slowly up the doses or never reached the higher doses.

All 60 patients experienced at least one AE during the study (Table 7) and a total of 464 AEs were reported. Twelve (20%) patients discontinued due to an AE; 7 patients discontinued on the 10 mg dose, 2 patients on the 20 mg dose and 3 patients on doses of 30 to 50 mg. Two patients experienced a serious AE (1-mild cataract, 1-severe ankle fracture), but these were not assessed as treatment-related by the investigators. The most common treatment-emergent AEs are presented in Table 8. At the 50 mg dose, the AE with the highest incidence was diarrhoea (5/31, 16%).

**Table 7:** Number (%) of patients reporting an adverse event, safety population

Category of AE	n = 60
Any adverse events (AEs)	60 (100)
Treatment-related AEs	
Possibly treatment-related	32 (53.3)
Probably treatment-related	16 (26.7)
Definitely treatment-related	0 (0)
Serious AEs	2 (3.3)
Serious treatment-related AEs ^a^	0 (0)
Discontinuations due to AEs ^b^	12 (20.0)
Treatment-related discontinuations due to AE ^a^	10 (16.7)

^a ^Number (%) of patients with an AE assessed as possibly, probably or definitely related to study medication

^b ^The AE case report form (CRF) page captured information regarding whether a patient withdrew from the study as a result of an AE. The End of Study CRF page captured withdrawal information and stated that a patient withdrew due to an AE, although this was not recorded as a withdrawal as a result of an AE on the AE CRF page

**Table 8:** Most common adverse events (reported by ≥10% of patients), total and by dose at onset of AE, safety population (n = 60)

	Number (%) of patients					
		**Dose at onset**				
**Preferred term**	**Total**	**10 mg**	**20 mg**	**30, 35 mg**	**40, 45 mg**	**50 mg**

Headache	21 (35.0)	10 (16.7)	6 (10.0)	5 (8.3)	1 (1.7)	3 (5.0)
Nausea	15 (25.0)	6 (10.0)	3 (5.0)	3 (5.0)	2 (3.3)	2 (3.3)
Diarrhoea	15 (25.0)	6 (10.0)	3 (5.0)	3 (5.0)	2 (3.3)	5 (8.3)
Nasopharyngitis	15 (25.0)	5 (8.3)	2 (3.3)	6 (10.0)	1 (1.7)	2 (3.3)
Hyperhidrosis	12 (20.0)	3 (5.0)	3 (5.0)	4 (6.7)	1 (1.7)	1 (1.7)
Upper respiratory tract infection	12 (20.0)	2 (3.3)	5 (8.3)	3 (5.0)	2 (3.3)	2 (3.3)
Fatigue	10 (16.7)	3 (5.0)	1 (1.7)	2 (3.3)	5 (8.3)	0 (0)
Dizziness	10 (16.7)	3 (5.0)	2 (3.3)	4 (6.7)	0 (0)	1 (1.7)
Vomiting	8 (13.3)	3 (5.0)	1 (1.7)	4 (6.7)	0 (0)	2 (3.3)
Abnormal dreams	6 (10.0)	2 (3.3)	0 (0)	3 (5.0)	1 (1.7)	1 (1.7)
Arthralgia	6 (10.0)	0 (0)	3 (5.0)	0 (0)	3 (5.0)	0 (0)
Back pain	6 (10.0)	0 (0)	2 (3.3)	2 (3.3)	1 (1.7)	1 (1.7)
Influenza like illness	6 (10.0)	1 (1.7)	1 (1.7)	1 (1.7)	1 (1.7)	3 (5.0)
Influenza	6 (10.0)	0 (0)	2 (3.3)	1 (1.7)	0 (0)	3 (5.0)
Lethargy	6 (10.0)	2 (3.3)	2 (3.3)	0 (0)	2 (3.3)	0 (0)
Oropharyngeal pain	6 (10.0)	0 (0)	2 (3.3)	2 (3.3)	1 (1.7)	1 (1.7)
Pain in extremity	6 (10.0)	0 (0)	2 (3.3)	3 (5.0)	2 (3.3)	0 (0)
Rash	6 (10.0)	2 (3.3)	2 (3.3)	0 (0)	1 (1.7)	2 (3.3)

**Figure 4:**
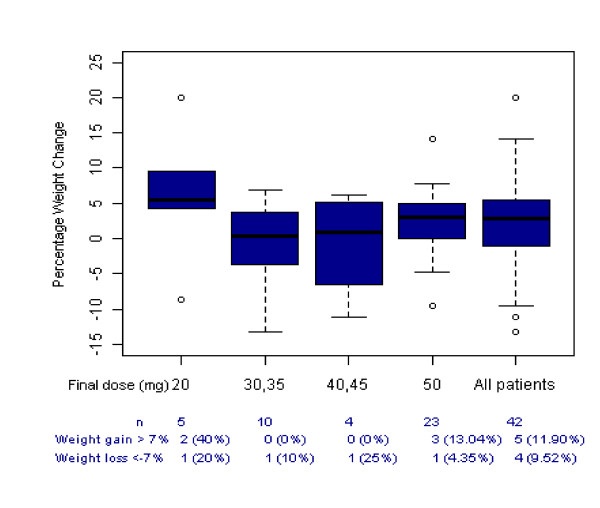
**Percentage weight change from baseline to end of study, by 32 week dose, completers (n = 42)**. The box and whisker plot shows the mean with upper and lower quartiles in the box and the whisker the minimum and maximum excluding outliers. Outliers are indicated by the small circles.

There was an increase of 0.83 kg (SD 4.86, n = 56) from baseline to the final visit in weight and 3.75 bpm (SD 10.90, n = 57) in the heart rate (safety population). Four (6.7%) patients reported weight gain as an AE and one patient discontinued treatment due to weight gain. The percentage weight gain is presented by dose at final visit for patients completing the study in Figure 4 and shows that weight gain did not appear to be dose related. Potentially clinically significant weight gain (>7%) was found in 12% of patients completing the study (though note that significant weight loss (>7%) was found in 10% of the same group).

Two patients had potentially clinically significant changes during the study. One patient, a 22 year-old female, with a normal ECG at baseline had an abnormal ECG (inverted T wave) at the end of the study, although this was assessed as not clinically significant by an independent assessor. Another female patient had a clinically significant blood test (raised γ-glutamyl transferase [GGT] 175 IU/L) at the end of the study, which was within the normal range at baseline (25 IU/L). After completing the study, the patient remained on escitalopram on the decision of her supervising clinician. Regular follow up assessments have shown that her GGT level is starting to revert towards normal.

**Figure 5:**
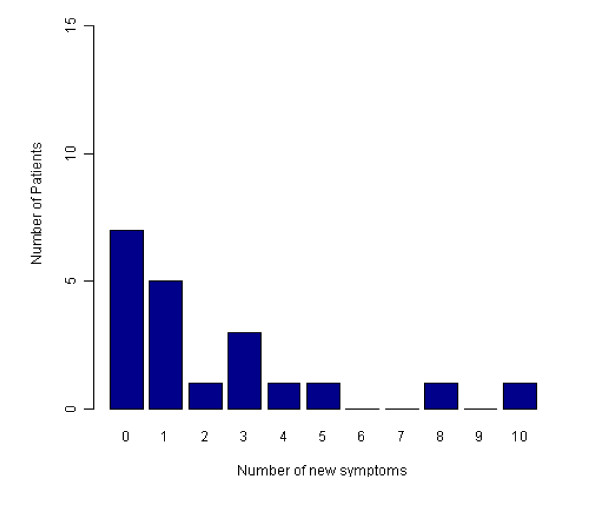
**Number of Discontinuation Emergent Signs and Symptoms reported by completers stopping treatment with escitalopram (n = 20)**. The number of new symptoms reported by the 20 patients who completed the study and tapered down the dose of escitalopram at 32 weeks prior to stopping treatment, as assessed using the Discontinuation Emergent Signs and Symptoms (DESS) scale scores.

Twenty of the 42 (48%) patients completed the study and tapered down the dose of escitalopram at 32 weeks prior to stopping treatment as given in the protocol. Twelve of these patients (60%) reported either no new symptoms or one new symptom on discontinuation, as assessed using the DESS scale scores (Figure 5). The most common new symptoms (≥15%) on stopping escitalopram were headache, dizziness, light-headedness or vertigo and muscle tension or stiffness.

## Discussion

This study showed that approximately one third of the patients who had not responded to treatment with at least 20 mg of citalopram for at least 6 weeks achieved remission (MADRS ≤8) during the 34 weeks of treatment with doses of escitalopram at doses up to 50 mg. A further third did not complete the study for a range of reasons including intolerance of escitalopram. Thus, half of those who did complete the study achieved remission.

Entry to the study was based on a minimum treatment with citalopram for six weeks, but neither dosage regime nor length of treatment was recorded. This and the difference in remission criteria make comparisons with outcomes in other studies such as STAR-D difficult. The patients could not strictly be described as treatment resistant and so comparison with the initial phase patients in STAR-D may be appropriate. In STAR-D, a remission rate of 28% as assessed by HAM-D ≤7 was achieved with a mean citalopram dose of 41.8 mg prescribed by physicians for up to 14 weeks. In this study, 40.8% of patients were in remission (MADRS ≤8) at 16 weeks following an accelerated dosage programme at a mean dose of 41 mg escitalopram.

Factors such as gender, age group and history of anxiety may influence outcome and although none of the associations were statistically significant in this study, these should be investigated in further studies.

Patients who responded at 8 weeks were significantly more likely to achieve remission at the end of the study. Therefore, a response to 20 or 30 mg at 8 weeks of this regime may be a useful predictor for achieving MADRS remission.

Doses up to 40 mg were generally well tolerated by patients in this study, with doses above this less well tolerated. Although 26% of all patients were unable to tolerate doses of 45 to 50 mg, 38% of the 21 patients who achieved remission needed the 50 mg dose.

There were no unexpected safety issues arising from the use of the higher doses of escitalopram in this study and only a small weight gain was observed, which did not appear to be dose related. Further studies are needed to establish the role of dose escalation of escitalopram in the management of patients with MDD who have not responded to conventional treatment with escitalopram.

Limitations of the study were the open nature of the study design, the small number of male patients and the high mean BMI of the study population (30.8), which may have influenced weight changes during treatment. No formal attempt was made to assess compliance during the study. The data have also been presented using OC and BOCF approaches. A last observation carried forward (LOCF) approach was not used to provide data for the 18 patients who discontinued the study. The reasons for this were that 6 patients withdrew after the first visit and had no available efficacy data and 9 patients had large fluctuations in the patterns of MADRS scores (results not shown). An LOCF approach could be justified for the remaining 3 patients who all withdrew after week 8, based on the MADRS pattern, although 2 of these withdrew due to AEs and one was ineligible to continue.

The data obtained from this pilot study might be used for the design of any subsequent clinical development programme. In the sample size calculations for this study, the assumptions of an attrition rate of 10% and a MADRS remission rate of 70% were not met and should be revised in any future trials. The observed attrition rate of 30% was considerably higher than expected, whereas the remission rate of 50% achieved was lower than expected. The high drop-out rate was mainly due to patients discontinuing due to AEs and this was particularly evident in the first two weeks of treatment. Consideration should be given to how the drop-out rate could be limited in future trials, possibly by improving patient awareness of the transient nature of some side effects including worsening of depression/anxiety/low mood on changing to a low dose of another medication.

## Conclusions

Dose escalation with escitalopram above 20 mg may have a useful role in the management of patients with treatment-resistant MDD, although larger randomised controlled studies are needed to confirm this finding.

## Competing interests

AGW has received consultancy fees from Lundbeck A/S, CreativCeutical, AstraZeneca, Pharmaneuroboost, Otsuka Pharmaceuticals, Lilly, Neurim Pharmaceuticals, Servier; lecture fees from Lundbeck A/S, Neurim and Pharmaneuroboost.

CPS Research of which GC and AGW are directors has received financial and research support from H. Lundbeck A/S, Pharmaneuroboost, Neurim, Wyeth, Pfizer and Servier.

GC and AY have no additional disclosures to make.

## Authors' contributions

AGW and GC designed the protocol, supervised the clinical work and collected the data. AY designed the statistical plan and was responsible for data entry and analysis. All three authors were involved in the production of the manuscript.

## Pre-publication history

The pre-publication history for this paper can be accessed here:

http://www.biomedcentral.com/1471-244X/11/42/prepub
